# Soluble fms-like tyrosine kinase 1, placental growth factor and procalcitonin as biomarkers of gram-negative sepsis: Analysis through a derivation and a validation cohort

**DOI:** 10.1097/MD.0000000000027662

**Published:** 2021-11-05

**Authors:** Vasileios Vittoros, Evdoxia Kyriazopoulou, Malvina Lada, Iraklis Tsangaris, Ioannis M. Koutelidakis, Evangelos J. Giamarellos-Bourboulis

**Affiliations:** a 1st Department of Internal Medicine, Thriasio General Hospital of Elefsis, G. Gennimatas Avenue, Athens, Greece; b 4th Department of Internal Medicine, National and Kapodistrian University of Athens, Medical School, 1 Rimini Street, Athens, Greece; c 2nd Department of Internal Medicine, Sismanogleion General Hospital of Athens, 37 Sismanogleiou Street, Athens, Greece; d 2nd Department of Critical Care Medicine, National and Kapodistrian University of Athens, Medical School, 1 Rimini Street, Athens, Greece; e 2nd Department of Surgery, Aristotle University of Thessaloniki, 41 Ethnikis Amynis street, Thessaloniki, Greece.

**Keywords:** diagnosis, procalcitonin, prognosis, sepsis, s-Flt-1

## Abstract

Further improvement of the diagnostic and prognostic performance of biomarkers for the critically ill is needed. Procalcitonin (PCT), placental growth factor (PlGF) and soluble fms-like tyrosine kinase-1 raise interest for sepsis diagnosis and prognosis.

Serum samples from 2 cohorts of 172 patients (derivation cohort) and of 164 patients (validation cohort) comprising only patients with microbiologically confirmed gram-negative infections were analyzed. PlGF, s-Flt-1 and procalcitonin (PCT) were measured in serum within 24 hours from sepsis onset and repeated on days 3 and 7.

PCT and s-Flt-1 baseline levels were higher in sepsis and septic shock compared to non-sepsis; this was not the case for PlGF. s-Flt-1 at concentrations greater than 60 pg/ml diagnosed sepsis with sensitivity 72.3% and specificity 54.9% whereas at concentrations greater than 70 pg/ml predicted unfavorable outcome with specificity 73.0% and sensitivity 63.7%. At least 80% decrease of PCT and/or PCT less than 0.5 ng/ml on day 7 was protective from sepsis-associated death.

Both s-Flt-1 and PCT should be measured in the critically ill since they provide additive information for sepsis diagnosis and prognosis.

ClinicalTrials.gov numbers NCT01223690 and NCT00297674.

## 1 Introduction

Although the global burden of sepsis may be underestimated due to lack of real data from low-income countries, it is generally considered that the global prevalence is over twenty million cases with mortality exceeding 20%.^[1]^ Early diagnosis may lead to early administration of antibiotics and this is the only available strategy that can effectively decrease mortality.^[2]^ However, in many cases the course of sepsis is subtle leading to delay of recognition and to subsequent unfavourable outcome.^[3]^ In this situation biomarkers remain the only tool that may contribute to early diagnosis. The diagnostic utility of procalcitonin (PCT) varies considerably between studies. In meta-analyses, pooled sensitivity and specificity do not exceed 85%.^[4–8]^ Two protein biomarkers, already used in embryo-fetal diagnostics, namely placental growth factor (PlGF) and soluble fms-like tyrosine kinase-1 (s-Flt-1) seem to attract much attention. PlGF is part of the vascular endothelial growth factor (VEGF) family^[9]^ and s-Flt-1 is a tyrosine kinase protein, a non-membrane VEGF receptor which binds to the angiogenic factors VEGF and PlGF. The physiologic role of s-Flt-1 remains unclear.^[10]^ Several prospective studies with small number of patients have shown that s-Flt-1 is increased in infections. This increase is pronounced among patients with sepsis caused by Gram-negative bacteria^[11–13]^ and it is even taking place in neutropenic patients.^[14]^ It is suggested that the increase of s-Flt-1 is sepsis is reciprocal to the increase of VEGF and it is aiming to counterbalance the action of VEGF.^[15]^

The current study investigated the potential of the combined information by these 3 biomarkers for the follow-up of sepsis using a derivation and a validation cohort. Our aim was to investigate if the overtime changes of these biomarkers may predict sepsis outcome and if their levels at disease onset may be diagnostic for sepsis and predictive of unfavorable outcome.

## 2 Materials and methods

### 2.1 Study population

The study population was coming from 2 randomized clinical trials (RCT) of the efficacy of intravenous clarithromycin for the management of sepsis. Patients of both original cohorts were recruited before 2012. The first RCT was conducted in 5 study sites in Greece during the period July 2007 to April 2011^[16]^ and was approved by the National Ethics Committee of Greece (approval 42797/20-06-2007), and the National Organization for Medicines of Greece (approval 76305/15-02-2007) (EudraCT number 2006-004886-33). Written informed consent was provided from all participants or their legal representatives before enrolment in the study. In this RCT, only patients with clinical or microbiologically proven Gram-negative infections aggravated by systemic inflammatory response syndrome (SIRS) could be enrolled (ClinicalTrials.gov identifier, NCT01223690). Serum was collected on day 1 (before start of blind treatment), 3, and 7. Since the 28-day mortality of patients allocated to intervention was similar for both arms, patients were analyzed together. Only samples coming from patients with microbiologically-confirmed Gram-negative infections were selected for this study. Samples were kept refrigerated at –80°C at the study central lab located at the 4^th^ Department of Internal Medicine. During the conduct of the study, levels of PCT were measured in patients; measurements were repeated in 2015 for the needs of the present study. It was found that measurement deviation was less than 5%.

Participants in the second RCT were patients with ventilator-associated pneumonia (VAP) aggravated by SIRS and they were enrolled during the period June 2004 to November 2005 (ClinicalTrials.gov identifier, NCT00297674).^[17]^ The study was approved by the National Organization for Medicines of Greece (approval 14653/14-6-2004) and was conducted according to the Declaration of Helsinki. Twenty-eight-day mortality of patients allocated to each arm did not differ and patients were analyzed together. Only samples coming from patients with microbiologically-confirmation for infections by Gram-negative bacteria were selected for this study; serum samples were kept at the same central lab under the same conditions. Biomarkers, including PCT, were measured in these patients during the conduct study; measurements were repeated in 2015 for the needs of the present study and deviation was less than 5%.

In both cohorts, patients were classified according to the Sepsis definitions of 1991.^[18]^ We merged the cohorts and we, then, randomly split them into a derivation cohort and a validation cohort at 1:1 ratio. The standard-of-care for these patients remained unchanged during the period of recruitment of both cohorts and it was based on the Surviving Sepsis Campaign guidelines.

KRYPTOR automated immunofluorescent assays (BRAHMS GmbH, Hennigsdorf, Germany) were used for the measurement of PCT, free PlGF and s-Flt-1. The lower detection limit of PCT was 0.02 ng/ml, PlGF 3.6 pg/ml, and s-Flt-1 22 pg/ml. All measurements were performed in duplicate and reported by technicians blinded to clinical information.

For the purposes of the study, percent changes of the levels of each biomarker on day 3 and 7 were calculated as *delta-biomarker (Δ*_*biomarker*_*)* *=* *(biomarker on day 1- biomarker on day 3 or 7)* ×*100/biomarker on day 1*. Changes of day 7 were further analyzed using the criterion for the change of PCT kinetics already proposed by de Jong et al^19^. More precisely, patients were split into 2 subgroups; those experiencing decrease of baseline PCT more than 80% or PCT less than 0.5 ng/ml by day 7; and those experiencing baseline PCT decrease less than 80% but maintaining their PCT more than 0.5 ng/ml.

The primary endpoint was to investigate the overtime changes of these 3 biomarkers and to detect the earliest timepoint the biomarker change may predict final outcome. Regarding PCT, this endpoint was also studied using the changes described above.^[19,20]^ Secondary endpoints were the baseline performance of these biomarkers for prediction of final outcome; and diagnosis of severe sepsis/septic shock.

### 2.2 Statistical analysis

Patients were classified as suffering from infection/SIRS and severe sepsis/septic shock. Categorical data were presented as frequencies and confidence intervals (CIs); continuous variables with normal distribution were expressed as means with standard error; variables with non-normal distribution were presented as boxplots. Fisher exact test was used for comparison of categorical data whereas Student *t* test or nonparametric Mann Whitney test were used for the comparison of parametric and nonparametric data respectively. Wilcoxon rank sum test was used for paired comparisons. Multivariable logistic regression was used to identify variables associated with higher mortality. The diagnostic or prognostic capacity of each biomarker was evaluated by the area under the respective receiver operating characteristics curve and the 95% CI. The optimal cut-offs were calculated by the Youden index. Survival comparisons were done by the log-rank test and comparisons of the odds ratio (OR) for death by the Tarone and Breslow-Day tests. Any 2-sided *P* value lower than.05 was considered statistically significant. Statistical analysis was performed using the software SPSS version 25.0 (IBM SPSS Statistics).

## 3 Results

**Figure 1:**
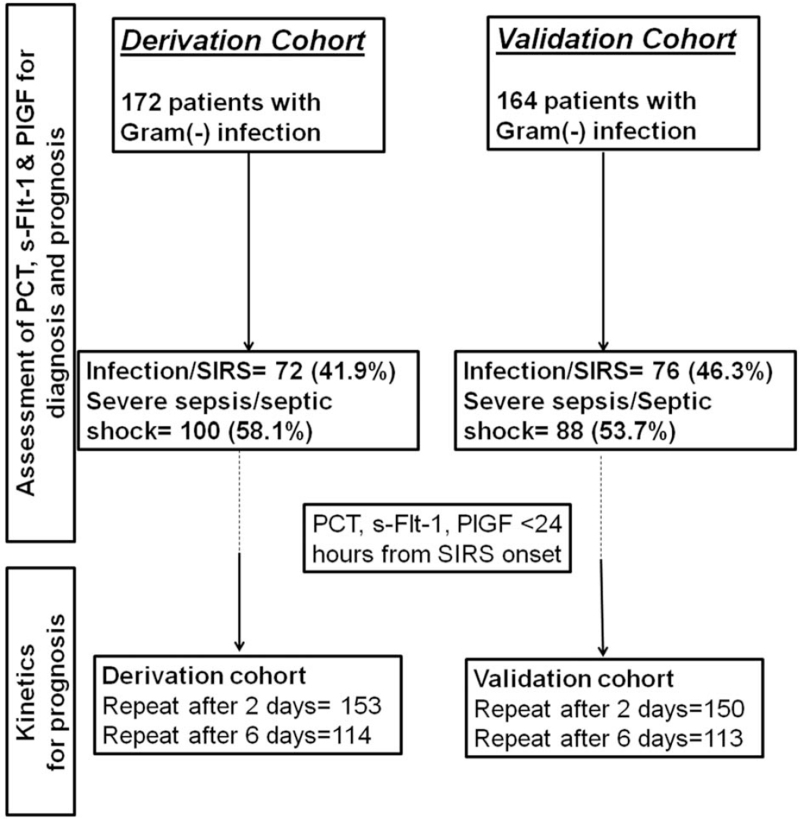
Study flow chart. PCT = procalcitonin, PlGF = placental growth factor, s-Flt-1 = soluble fms-like tyrosine kinase-1, SIRS = systemic inflammatory response syndrome.

The study flow chart is shown in Figure 1; 172 patients were included in the derivation cohort and 164 in the validation cohort. Baseline demographics did not differ between the cohorts (Table 1).

**Table 1:** Baseline characteristics of patients enrolled in the study.

	Derivation cohort (N = 172)	Validation cohort (N = 164)	*P*
APACHE II, mean ± SD	14.8 ± 7.1	15.0 ± 7.1	.785
SOFA, mean ± SD	4.0 ± 3.9	3.8 ± 4.1	.704
Age (yrs), mean ± SD	65.3 ± 19.5	65.5 ± 19.0	.944
Charlson comorbidity index, mean ± SD	3.5 ± 2.4	3.4 ± 2.3	.886
Type of infection, n (%)			
Acute pyelonephritis	63 (36.6)	64 (39.0)	.655
Intraabdominal infection	21 (12.2)	18 (11.0)	.737
Primary bacteremia	33 (19.2)	33 (20.1)	.891
Ventilator associated pneumonia	55 (32.0)	49 (29.9)	.724
Isolated pathogens, n (%)			
*Escherichia coli*	58 (33.7)	52 (31.7)	.728
*Αcinetobacter baumannii*	40 (23.3)	32 (19.5)	.427
*Klebsiella pneumoniae*	18 (10.5)	23 (14.0)	.405
*Pseudomonas aeruginosa*	21 (12.2)	19 (11.6)	.868
*Enterobacter cloacae*	0 (0.0)	7 (4.3)	**.006**
*Proteus mirabilis*	7 (4.1)	8 (4.9)	.795
*Providentia stuartii*	2 (1.2)	2 (1.2)	1.000
*Enterobacter aerogenes*	0 (0.0)	1 (0.6)	.488
*Stenotrophomonas maltophilia*	3 (1.7)	2 (1.2)	1.000
Other Gram-negative	16 (9.3)	11 (6.7)	.427
Polymicrobial Gram-negative infection	7 (4.1)	5 (3.0)	.771

In bold, characteristics that differed significantly between the 2 cohorts. APACHE = acute physiology and chronic health evaluation, SD = standard deviation, SOFA = sequential organ failure assessment.

**Figure 2:**
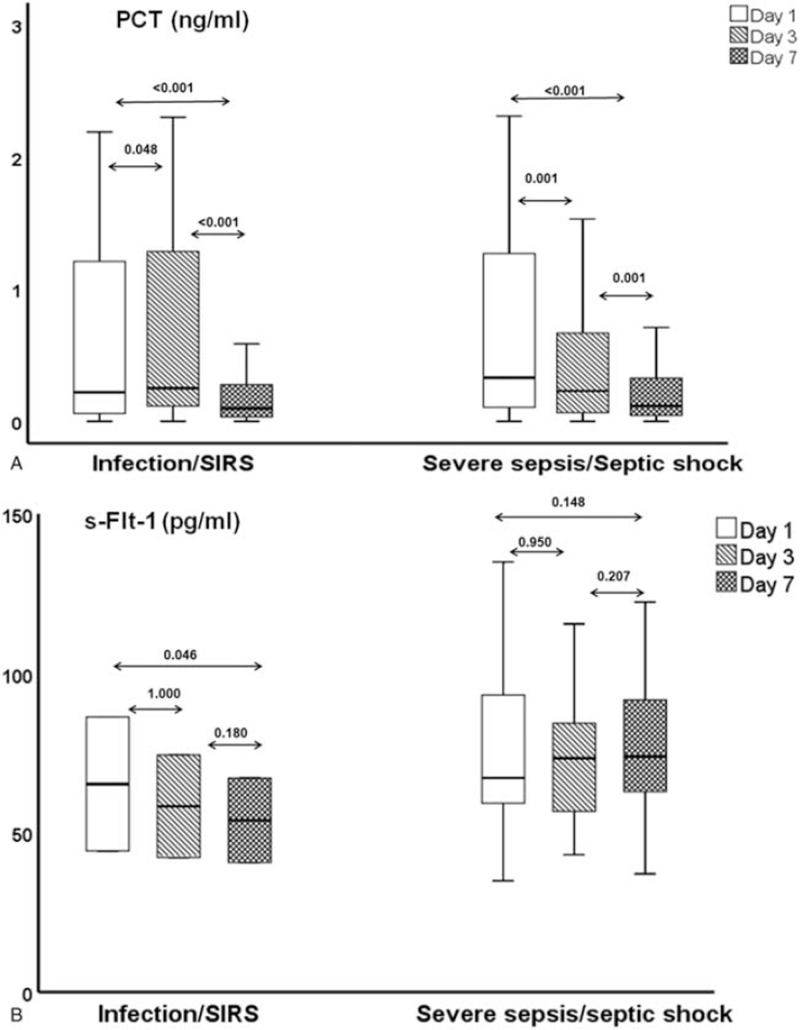
Overtime changes of biomarkers in the derivation cohort. Serum concentrations of procalcitonin (PCT) (A) and of soluble fms-like tyrosine kinase-1 (s-Flt-1) (B) on days 1 (baseline), 3, and 7. *P* values of the indicated comparisons are provided. Comparisons are done by the Wilcoxon rank sum test.

**Figure 3:**
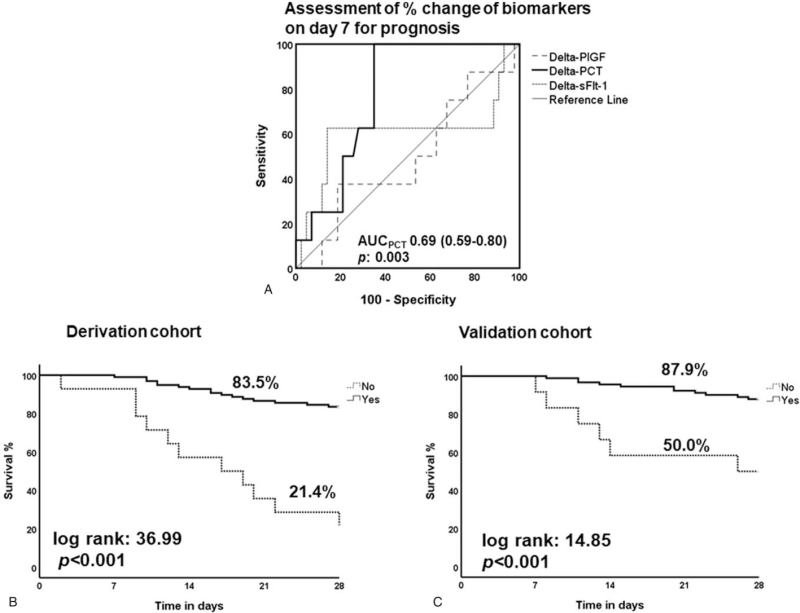
Serum procalcitonin (PCT) as surrogate marker of final outcome. (A) ROC curves of percent change of serum procalcitonin (PCT), of placental growth factor (PlGF) and of soluble fms-like tyrosine kinase-1 (s-Flt-1) between baseline day 1 and 7 for the prognosis of unfavorable outcome; AUC = area under the curve. Kaplan-Meier curves of survival in the derivation (B) and (C) validation cohorts. Yes and No applies to meeting or not meeting the following criteria of kinetic change on day 7: at least 80% decrease of PCT and/or PCT<0.5 ng/ml. Log-rank tests and *P* values of significance are provided.

The concentrations of PCT, but not of s-Flt-1, were decreasing over follow-up (Fig. 2). The decrease of PCT on day 7 was associated with favorable outcome (Fig. 3A). When survival was compared between patients experiencing baseline decrease of PCT more than 80% or maintaining PCT less than 0.5 ng/ml by day 7 and patients experiencing baseline decrease of PCT less than 80% and maintaining PCT more than 0.5 ng/ml by day 7, differences were profound in both the derivation (Fig. 3B) and the validation cohorts (Fig. 3C). This was further confirmed following logistic regression analysis where either more than 80% decrease of PCT and/or any PCT value lower than 0.5 ng/ml was associated with favorable outcome after adjustment for illness severity, presence of comorbidities, body mass index, mechanical ventilation, appropriateness of administered antimicrobials and source control (OR 0.03; 95% CI: 0.04-0.26) (Table 2).

**Table 2:** Univariable and multivariable logistic regression analysis of predictors of 28-d outcome.

	Univariable analysis			Multivariable logistic regression analysis		
	OR	95% CI	*P*	OR	95% CI	*P*
APACHE II	1.17	1.10-1.25	<.001	1.03	0.93-1.14	.582
Charlson comorbidity index	1.75	0.92-3.35	.089	**1.54**	**1.08-2.20**	**.018**
Body mass index > 30	0.74	0.08-6.51	.785	0.95	0.03-35.63	.976
Inappropriate antimicrobial treatment	1.65	0.48-5.74	.429	0.89	0.08-10.18	.925
Source control	0.69	0.19-2.50	.570	0.47	0.06-3.43	.455
Mechanical ventilation	4.46	2.09-9.52	<.001	2.11	0.42-10.53	.361
Presence of shock	8.80	4.34-17.83	<.001	**7.23**	**1.34-38.94**	**.021**
≥80% baseline decrease of PCT or PCT <0.5 ng/ml on day 7	0.05	0.01-0.22	<.001	**0.03**	**0.01-0.26**	**.001**

In bold, variables that were associated significantly with 28-d outcome after adjusted multivariable (logistic regression) analysis. APACHE = acute physiology and chronic health evaluation, CI = confidence interval, OR = odds ratio, PCT = procalcitonin.

**Figure 4:**
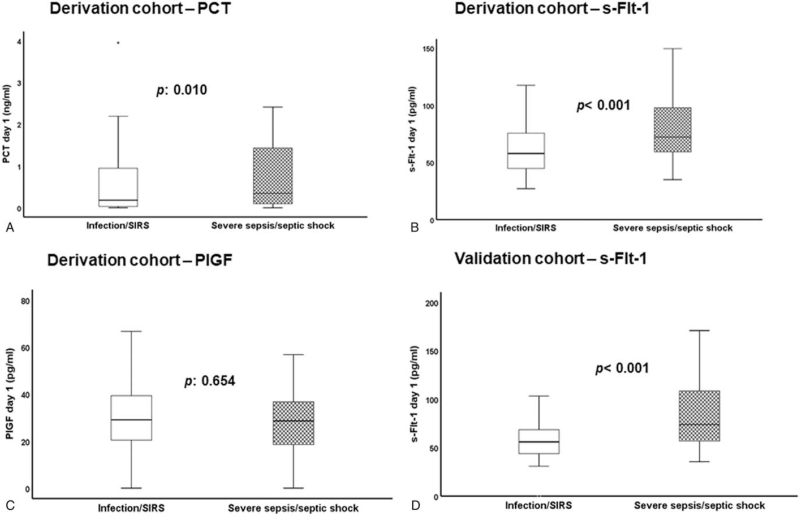
Biomarkers for the diagnosis of sepsis. Derivation cohort: comparison of serum levels of (A) procalcitonin (PCT); (B) soluble fms-like tyrosine kinase-1 (s-Flt-1); and (C) placental growth factor (PlGF) between patients with infection/SIRS (systemic inflammatory response syndrome) and patients with severe sepsis/septic shock. Validation cohort: comparison of serum levels of s-Flt-1 (D) between patients with infection/SIRS and patients with severe sepsis/septic shock. *P* values of comparisons are indicated by the arrows.

**Figure 5:**
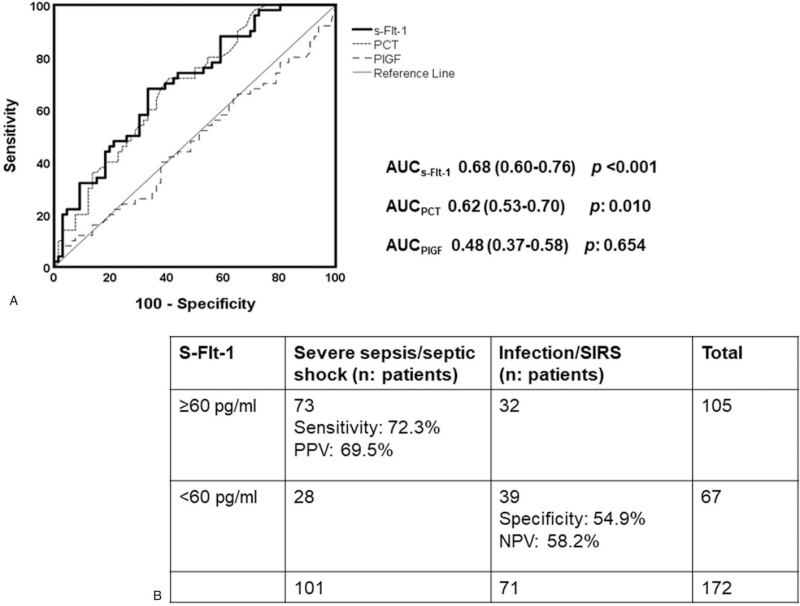
Diagnostic cut-off of s-Flt-1 in the derivation cohort. (A) ROC curve of procalcitonin (PCT), placental growth factor (PlGF) and soluble fms-like tyrosine kinase-1 (s-Flt-1) for the diagnosis of severe sepsis/septic shock; AUC = area under the curve. (B) Sensitivity, specificity, positive predictive value (PPV) and negative predictive value (NPV) of serum s-Flt-1 above 64 pg/ml for the diagnosis of severe sepsis/septic shock.

Regarding the secondary endpoint of sepsis diagnosis, PCT, and s-Flt-1 on the first day were higher in patients with severe sepsis/septic shock compared to infection/SIRS; PlGF could not discriminate between infection/SIRS and severe sepsis/septic shock (Fig. 4). Using the Youden index of the ROC curve analysis (Fig. 5A), an optimal diagnostic cut-off of s-Flt-1 for severe sepsis/septic shock of 60 pg/ml was found. Values above this cut-off could discriminate severe sepsis/septic shock with sensitivity 72.3% and specificity 54.9% (Fig. 5B).

**Figure 6:**
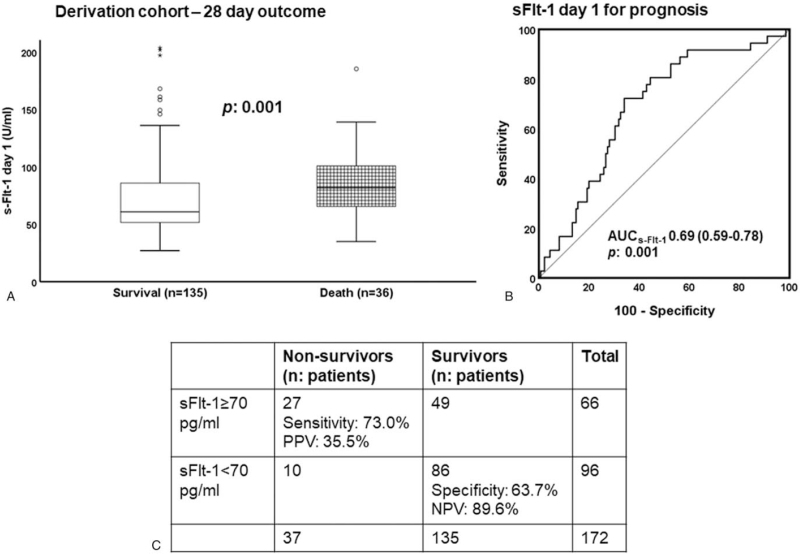
Development of biomarkers for the prognosis of sepsis in the derivation cohort. (A) Comparison of serum levels of soluble fms-like tyrosine kinase-1 (s-Flt-1) between survivors and non-survivors; (B) ROC curve of soluble fms-like tyrosine kinase-1 (s-Flt-1) for the prognosis of unfavorable outcome; AUC = area under the curve; (C) Sensitivity, specificity, positive predictive value (PPV) and negative predictive value (NPV) of serum s-Flt-1 above 70 pg/ml for the prognosis of unfavorable outcome; *P* values are provided.

**Figure 7:**
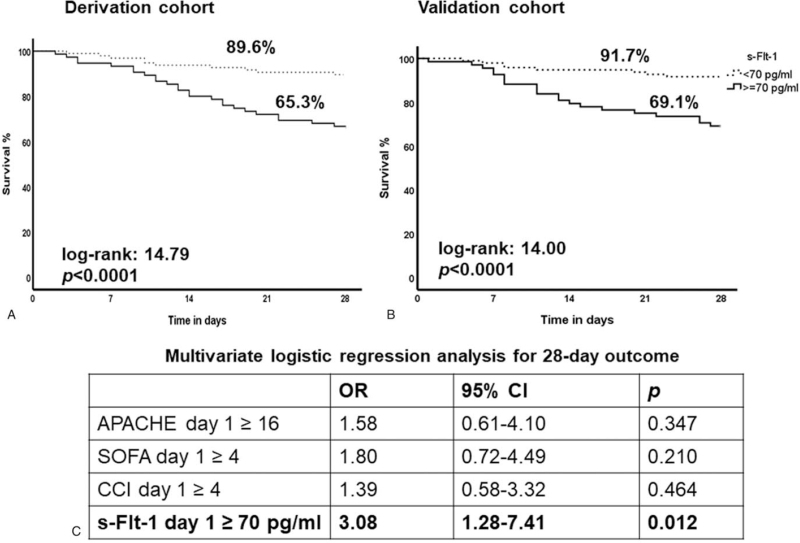
Validation of soluble fms-like tyrosine kinase-1 (s-Flt-1) for outcome prediction. Kaplan-Meier curves of survival in the derivation (A) and validation cohorts (B). Log-rank tests and *P* values are provided. (C) Multivariable logistic regression analysis of predictors of 28-d outcome. Cut-off values were calculated by the Youden index of the respective ROC curves for prediction of 28-d mortality. *P* values are provided. APACHE = acute physiology and chronic health evaluation, CCI = Charlson comorbidity index, CI = confidence interval, OR = odds ratio, s-Flt-1 = soluble fms-like tyrosine kinase-1, SOFA = sequential organ failure assessment.

PCT and PlGF levels at baseline could not predict 28-day outcome (data not shown). On the contrary, s-Flt-1 was higher among non-survivors (*P*:.001) (Fig. 6A). Using the Youden index of the ROC curve analysis (Fig. 6B), an optimal cut-off of s-Flt-1 for prognosis of 70 pg/ml was found. Values above this cut-off provide 89.6% negative predictive value for unfavorable outcome (Fig. 6C). Survival analysis confirmed this prediction cut-off point for both cohorts (Fig. 7A and B). Regression analysis confirmed that s-Flt-1 greater than 70 pg/ml was an independent predictor of unfavorable outcome (Fig. 7C).

**Figure 8:**
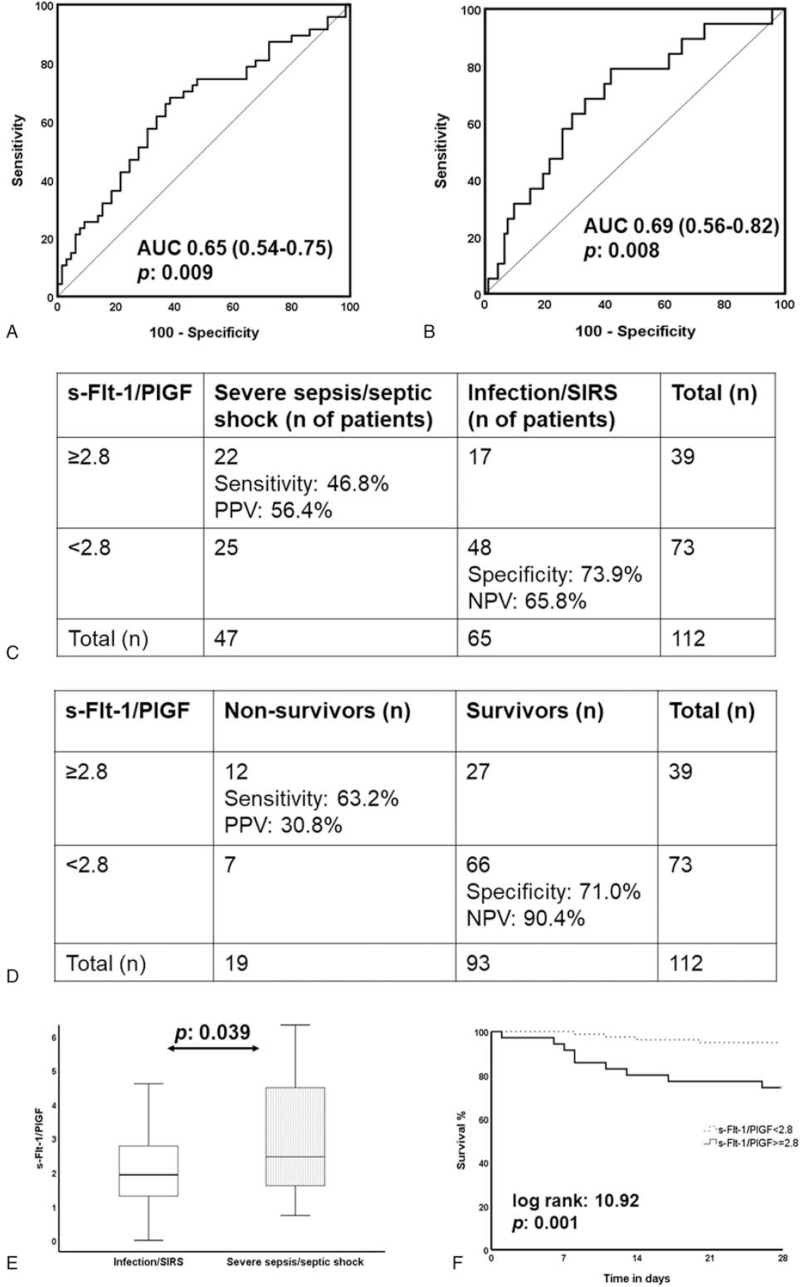
Development of soluble fms-like tyrosine kinase-1/placental growth factor ratio (s-Flt-1/PlGF) for the diagnosis and prognosis of sepsis in the derivation cohort. ROC curve of s-Flt-1/PlGF for (A) the diagnosis and (B) prognosis of severe sepsis/septic shock; AUC = area under the curve. Sensitivity, specificity, positive predictive value (PPV) and negative predictive value (NPV) of s-Flt-1/PlGF above 2.8 for (C) the diagnosis of severe sepsis/septic shock; and (D) the prognosis of final outcome. (E) Validation cohort: comparison of serum levels of s-Flt-1/PlGF between patients with infection/SIRS (systemic inflammatory response syndrome) and patients with severe sepsis/septic shock. (F) Kaplan-Meier curves of survival between patients with s-Flt-1/PlGF ≥2.8 or less than 2.8. The value of the log-rank test and the *P* value of comparison are provided.

Analysis also showed that the s-Flt-1/PlGF ratio at baseline, as investigated in other conditions such as preeclampsia, may provide additional help. The ratio is greater among patients at severe sepsis/septic shock (Fig. 8A) and values ≥2.8 may contribute to the diagnosis of severe sepsis/septic shock (area under the curve [AUC] 0.65; 95% CI, 0.54-0.75; *P*:.009; Fig. 8). Values ≥2.8 may also predict 28-day outcome (AUC 0.69; 95% CI, 0.56-0.82; *P*:.008; Fig. 8C) and they are associated with worse outcome (Fig. 8D). Sensitivity for the diagnosis of severe sepsis/septic shock above this cut-off was 46.8%; it was 63.2% for the prognosis of unfavorable outcome; specificity was 73.9% and 71.0%, respectively (Fig. 8E and F).

## 4 Discussion

Our study adds considerably to the current knowledge for sepsis biomarkers. It introduces baseline s-Flt-1 as an early diagnostic and prognostic tool since concentrations above 60 pg/ml can trace severe sepsis/septic shock whereas values above 70 pg/ml are associated with unfavorable 28-day outcome. Moreover, the current study further enlightens the value of PCT as the most reliable surrogate tool for follow-up; decrease by at least 80% or values less than 0.5 ng/ml are associated with favorable prognosis.

Our results corroborate data from 2 other cohorts of 161 and 170 patients. In the first cohort, 161 patients were admitted in the Emergency Department with hypotension; s-Flt-1 could detect those patients with sepsis as the cause of hypotension (OR adjusted 2.0; 95% CI: 1.1-3.8).^[21]^ In the second cohort of 170 sepsis patients, serum s-Flt-1 levels were significantly higher in patients with positive blood cultures than in patients with sterile blood cultures (277.7 ± 52.7 and 133.4 ± 12.4 pg/ml, respectively); they were also higher when the isolated pathogens were gram-negative bacteria (274.1 pg/ml vs 145.7 pg/ml for Gram-positive bacteria).^[12]^

The values of PCT on day 1 are lower than expected compared to other publications.^[22–25]^ This may reflect the study population. Despite this discrepancy, findings confirmed that at least 80% decreases from baseline and/or values less than 0.5 ng/ml over-time are associated with favorable prognosis.

In a subpopulation of the ProCESS trial comparing 3 different strategies of fluid resuscitation in sepsis, s-Flt-1 levels at baseline and for the first 24 hours from sepsis onset were related to mortality. The analysis was done after adjustment for age, presence of cancer and Charlson comorbidity index. More precisely, baseline was the time point where s-Flt-1 presented with AUC 0.74 performing similar to lactate and sequential organ failure assessment (SOFA) score; in the analysis of all time points and after adjustment for baseline variables the AUC was increased to 0.80.^[26]^ Our differences compared to the ProCESS trial are the enrolment of patients without shock, the suggestion of specific cut-offs for diagnosis and prognosis and the description of over-time changes. The different kinetics of s-Flt-1 between sepsis and septic shock may result from the most prominent damage of the vasculature in shock.

The diagnostic performance of PlGF was not satisfactory despite the results of previous animal and human studies.^[27,28]^ The injection of lipopolysaccharide (LPS) in animals caused substantial increase of PlGF in the circulation; this did not happen in animals with genetic absence of PlGF or after administration of neutralizing anti-PlGF antibodies.^[15]^

The main limitations of our study are the retrospective analysis of prospectively recruited patients and the retrospective processing of biological samples. Patient recruitment was performed before introduction of the Sepsis-3 definitions in 2016.

## 5 Conclusions

Current data suggest that s-Flt-1 and PCT provide additive information in the diagnosis and prognosis of sepsis regarding the follow-up of the patient. Information provided from the combination of both biomarkers acts synergistically and provides complete information both for diagnosis and prognosis. Validation of these results in larger prospective studies enrolling patients meeting the Sepsis-3 definition is needed.

## Author contributions

VV, ML, IT, IMK contributed to the collection of the data, revised the manuscript critically for important intellectual content and gave approval of the version to be published.

EK contributed to the analysis of the data, drafted the manuscript and gave final approval of the version to be published.

EJGB conceptualized the study design, contributed to the analysis of the data, participated in drafting the manuscript, critically reviewed the manuscript and gave final approval of the version to be published.

**Conceptualization:** Evangelos Giamarellos-Bourboulis.

**Data curation:** Vasileios Vitoros, Malvina Lada, Iraklis Tsangaris, Ioannis Koutelidakis.

**Formal analysis:** Evdoxia Kyriazopoulou, Evangelos Giamarellos-Bourboulis.

**Funding acquisition:** Evangelos Giamarellos-Bourboulis.

**Methodology:** Evangelos Giamarellos-Bourboulis.

**Project administration:** Evangelos Giamarellos-Bourboulis.

**Supervision:** Malvina Lada, Iraklis Tsangaris, Ioannis Koutelidakis.

**Validation:** Iraklis Tsangaris.

**Writing – original draft:** Evdoxia Kyriazopoulou.

**Writing – review & editing:** Evangelos Giamarellos-Bourboulis.
